# Single-incision laparoscopic surgery for jejuno-jejunal intussusception caused by an angiolipomatous polyp in an adult: A case report

**DOI:** 10.1097/MD.0000000000018280

**Published:** 2019-12-16

**Authors:** Young-Kyu Kim

**Affiliations:** Department of Surgery, Jeju National University School of Medicine, Jeju-si, Jeju Special Self-Governing Province, Republic of Korea.

**Keywords:** angiolipomatous polyp, jejuno-jejunal intussusception, single-incision laparoscopic surgery

## Abstract

**Rationale:**

Small bowel intussusception in adults is rare but is more likely to occur in the presence of a lead point. Surgical intervention is necessary in most cases, even if there is successful nonsurgical reduction of the intussusception.

**Patient concerns:**

A 54-year-old woman who was transferred to our emergency room with complaints of intermittent cramping pain of 4 days’ duration.

**Diagnosis:**

Abdominal contrast-enhanced computed tomography revealed a jejuno-jejunal intussusception due to an angiolipomatous polyp.

**Intervention:**

A single-incision laparoscopic surgery (SILS) was performed without the need for any additional incisions.

**Outcomes:**

She was uneventfully discharged on postoperative day 4.

**Lessons:**

The SILS procedure with adequate preoperative diagnosis by CT, with or without US, can offer good clinical outcomes for small bowel intussusception. Even surgeons who have little experience with laparoscopic intestinal anastomosis can consider SILS to treat small bowel intussusception in adults.

## 1 Introduction

Intestinal intussusception is defined as the telescoping of a segment of the gastrointestinal tract within the lumen of adjacent bowel.^[1]^ Most intussusceptions occur in childhood. Adult intussusception is rare, but usually occurs in association with a lead point. Benign or malignant tumors such as submucosal fibromas, lipomas, Meckel's diverticula, adenocarcinomas, and gastrointestinal stromal tumors are potential lead points for intussusception.^[1–4]^ However, intussusception caused by a single angiolipomatous polyp is extremely rare. We recently treated an adult patient with jejuno-jejunal intussusception caused by a single angiolipomatous polyp using a single-incision laparoscopic surgery (SILS).

## 2 Case report

A 52-year-old woman was transferred to our emergency room with complaints of intermittent cramping and mid-epigastric and left-upper-quadrant abdominal pain of 4 days’ duration. The abdominal pain was associated with nausea and lasted for a day with increasing intensity. She reported normal appetite and no weight loss within the previous year. She also had a 1-year history of recurrent dyspepsia. Therefore, gastroscopy and colonoscopy were performed, the findings of which were unremarkable. She did not undergo abdominal and pelvic ultrasound (US) or gynecological evaluation at this point. She underwent an open appendectomy 5 years previously at another hospital.

**Figure 1:**
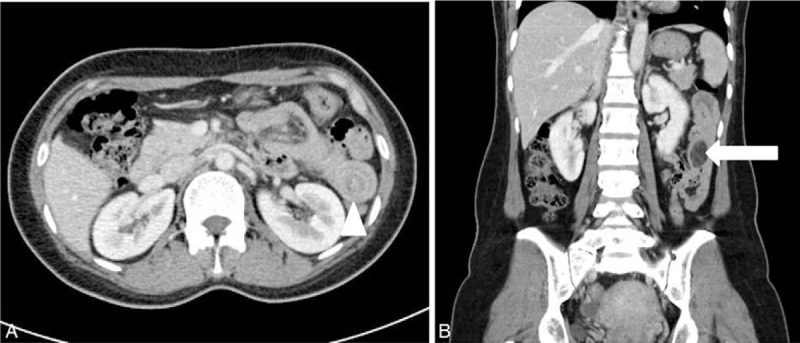
An axial abdominal contrast-enhanced computed tomography (CT) image shows dilated loop of small bowel consistent with a possible small bowel obstruction and a potential target sign (white arrow head) in the left upper quadrant (A). A coronal CT image showing a well-defined, low-fat-density mass (white arrow) (B), which we assumed was the lead point for the intussusception.

On clinical examination, her abdomen was soft and mildly distended, and she did not exhibit rebound tenderness. All laboratory findings were within normal ranges except for C-reactive protein (1.62 mg/dL). Her chest and abdominal radiography results were unremarkable. Her symptoms continued for another day, and metallic bowel sounds were audible by this time. Hence, we performed abdominal contrast-enhanced computed tomography (CT) which revealed a thickened loop of small bowel containing vascular elements and a target sign highly suggestive of jejuno-jejunal intussusception. CT also revealed the lead point of the intussusception (Fig. 1A). She was immediately taken to the operating theater for emergency surgery.

**Figure 2:**
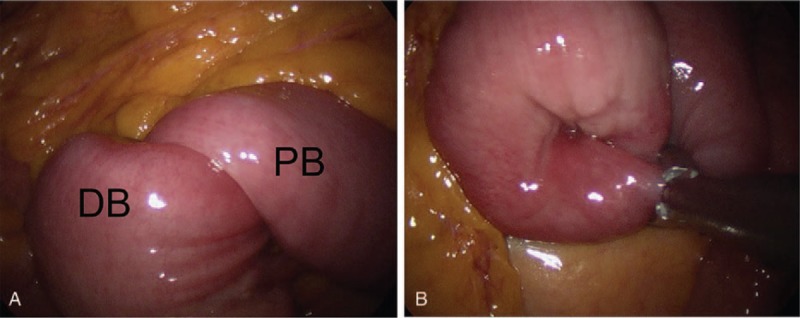
There were 2 laparoscopic views: one was just before the laparoscopic reduction (A) and the other was just after the laparoscopic reduction (B). DB = distal bowel, PB = proximal bowel.

A single ∼20 mm incision was made for SILS at the umbilicus. The jejuno-jejunal intussusception was visualized approximately 80 cm distal to the ligament of Treitz. The intussuscepted bowel segment was approximately 20 cm long. The involved bowel was dilated, but there was no evidence of bowel ischemia or perforation (Fig. 2A). An intracorporeal reduction of the intussusception was cautiously performed using atraumatic graspers according to the intracorporeal reduction technique reported by Siow and Mahendran.^[5]^ The atraumatic grasper was applied transversely across the whole length of the bowel diameter proximal to the apex of the intussusceptum, while another atraumatic grasper was applied on the distal bowel. The force was applied to both the graspers using the traction-counter traction technique (Fig. 2B). The surgeon checked the entire small bowel via the single laparoscopy port and noted a non-specific polypoid abnormality. He then extended the incision by 1 cm in both vertical directions to exteriorize the small bowel.

**Figure 3:**
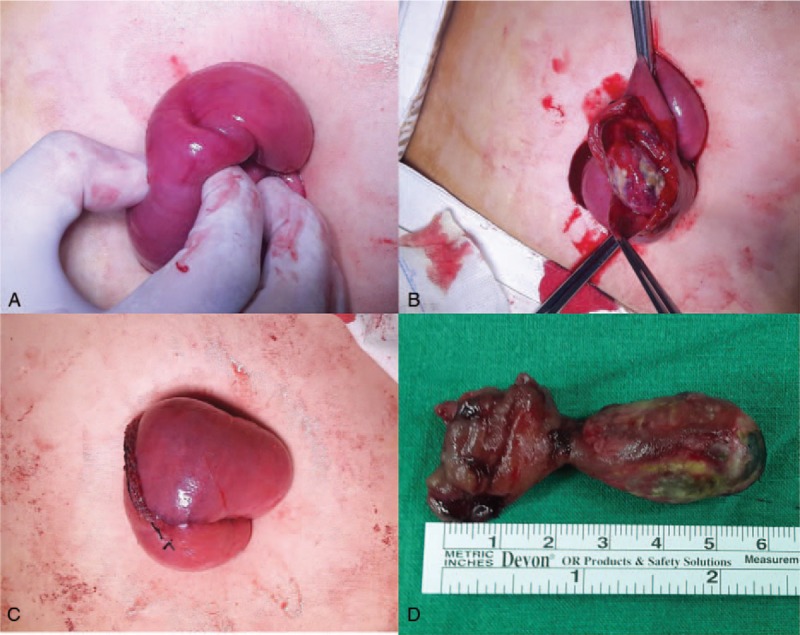
The involved small bowel was taken out via a small umbilical incision. When the polyp was pulled inferiorly, a dimple was created (A). The polyp was visualized after a longitudinal incision was made above it (B). The intestinal anastomotic site just after a segmental resection of small bowel and anastomosis were performed using surgical stapling devices (C). Gross image shows the 3 cm × 2.5 cm polyp in the jejunum (D).

**Figure 4:**
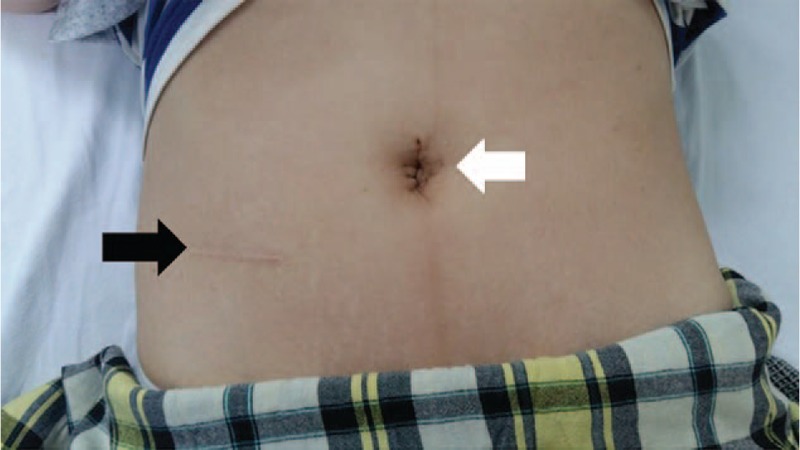
Postoperative picture showing a small, ∼3 cm long umbilical incision (white arrow). A scar due to a previous open appendectomy was observed (black arrow).

A luminally protruding polyp was palpable (Fig. 3A), which the surgeon supposed was the lead point for the intussusception (Fig. 3B). Approximately 1.5 cm of jejunum was resected, including the polyp. A side-to-side anastomosis was performed using the surgical stapling method (Fig. 3C). The anastomosed jejunum was placed back into the peritoneal cavity. The incision site was closed without inserting a drain. The total operation time was 65 minutes, and the estimated blood loss was 20 mL. The pathologic report indicated an angiolipomatous polyp, with a maximum diameter of 2.5 cm (Fig. 3D) and fatty cut surfaces. The postoperative course was uneventful, and the patient was discharged on the fourth postoperative day without complications. She visited the outpatient clinic on the eleventh postoperative day. With her consent, we photographed the wound (Fig. 4). The patient has also provided informed consent for publication of this report.

## 3 Discussion

Intussusception is classified into 4 types according the location of the leading point—the colocolic, ileocolic, ileocecal, and enteroenteric. The high incidence of tumors and the high activity of the small intestine makes the enteroenteric type of intussusception most common in adults. The enteroenteric type accounted for 45% to 73% of all adult intussusception.^[4,6,7]^ Malignant tumors caused approximately 30% of enteroenteric intussusception, whereas adenocarcinoma mainly caused the other types of adult intussusception.^[1,4,6–8]^ A study of adult intussusceptions in Korea showed that 58% of adult intussusception was the enteroenteric type, and 20% of the enteroenteric type was caused by malignancy.^[9]^

The optimal management of adult intussusception remains controversial, because most such intussusceptions are associated with a lead point, which could potentially be malignant. It is still debated whether en bloc resection is superior to initial reduction followed by less-extensive resection to avoid bowel perforation and seeding of potential cancer cells.^[4,6–8]^ Recently, colonoscopy and CT, with or without US, have helped rule out the possibility of malignancy to facilitate adequate and successful reduction and accurate diagnosis,^[7]^ making minimal resection of the involved bowel a possibility. Fortunately, inflammatory conditions and benign tumors are the more likely causes of adult small bowel intussusception; however, this is not the case with colonic intussusceptions. In either case, CT with or without US helps characterize the lead point to guide the choice between laparoscopy and conventional open surgery for adult intussusception cases. The advantages of laparoscopic surgery include its minimal invasiveness and association with less pain and faster recovery. Surgeons should be encouraged to treat adult intussusception laparoscopically if possible.

Conventional laparoscopic surgery is widely used instead of open surgery for acute abdominal diseases. Moreover, some authors reported the feasibility and safety of laparoscopy in the diagnosis and treatment of adult intussusceptions.^[5,10]^ Recently, SILS—a further less invasive procedure—has been indicated for various conditions.^[11,12]^ However, SILS has technical disadvantages compared to conventional laparoscopic surgery. First, counter-traction by an assistant is impossible, because only 2 forceps can be simultaneously used by the surgeon. Second, surgical manipulations are restricted because of the interference with the forceps and scope.^[13]^ Nevertheless, SILS is primarily attractive given the usefulness of a relatively big single incision. SILS can observe the entire small intestine, guide intussuscepted bowel which cannot be laparoscopically reduced outside of the body via a planned or an extended incision, and quickly perform an extracorporeal resection and anastomosis of the small intestine after potentially manual reduction without any additional incision.^[11]^ Creation of additional incisions or an extended incision is rarely needed in SILS for enteroenteric intussusception, because the umbilical port hole can be used as a window for extracorporeal procedures, if reduced intracorporeally. Siow and Mahendran asserted that an extracorporeal rather than intracorporeal resection and anastomosis should be attempted as enteroenteric intussusceptions of any length could be exteriorized through a 3 to 4 cm incision.^[5]^

Laparoscopically intracorporeal reduction is difficult in patients with long length (>50 cm) or severe edema of intussuscepted bowel and is contraindicated in patients with a malignant leading point or bowel ischemia.^[5]^ An extracorporeal resection and anastomosis of the small bowel with a manual reduction in the former patients or without a manual reduction in the latter (via an extended incision) can not only prevent additional bowel injuries or dissemination of cancer cells but also decrease operation time and procedure-associated complications in those patients.^[5]^

Surgical strategy for adult intussusception can be tailored according to the type of intussusception, possibility of underlying disease, and availability of surgeons with laparoscopic expertise.^[4]^ A reduction before resection may afford only limited resection, but the reduction of the intussuscepted bowel may cause seeding of potential tumor cells and microorganisms in the abdominal cavity, bowel perforation, and increased anastomotic leakage.^[4,6–8]^ Thus, in patients with ileocolic, ileocecal, and colocolic intussusceptions, formal resections using appropriate oncologic techniques are recommended, particularly in those >60 years old, given the high incidence of bowel malignancy as the underlying etiologic factor.^[4]^ Therefore, open or conventional laparoscopic surgery by only surgeons with laparoscopic expertise is indicated in those patients. SILS is contraindicated in them because of its limitations such as poor count-traction by an assistant and limited surgical manipulations. However, in patients with enteroenteric intussusception, SILS can be unquestionably indicated irrespective of surgeons’ laparoscopic expertise or underlying disease, because the role of laparoscopy in this type of intussusception is different from that in other types. The small bowel is freely attached by the mesentery to the posterior abdominal wall, and it can be easily exteriorized without any dissections. Thus, the role of laparoscopy for enteroenteric intussusception is to localize a lesion and guide it to be exteriorized via a planned or an extended incision to perform extracorporeal procedures such as a resection, anastomosis of small bowel, or manual reduction. SILS performed by surgeons who have relatively little laparoscopic experience or skill can safely be indicated in patients with enteroenteric type of adult intussusception. In our report, even though the surgeon had minimal training in laparoscopic techniques, he completed the procedure in 65 minutes without any difficulties, and the patient recovered without any complications.

## 4 Conclusion

This was a rare case of an angiolipomatous polyp in the jejunum causing jejuno-jejunal intussusception in an adult, which was treated via SILS. Imaging modalities such as CT, with or without US, can help to characterize the intussusception. The potential of malignancy is a concern in the context of adult intussusception, and treatment strategies remain controversial. A laparoscopic approach with adequate preoperative diagnosis by CT, with or without US, can offer good clinical outcomes. Even surgeons who have limited experience with laparoscopic intestinal anastomosis can consider SILS to treat small bowel intussusception in adults.

## Author contributions

**Conceptualization:** Young-Kyu Kim.

**Methodology:** Young-Kyu Kim.

**Writing – original draft:** Young-Kyu Kim.

**Writing – review & editing:** Young-Kyu Kim.
